# Why Iranian married women use withdrawal instead of oral contraceptives? A qualitative study from Iran

**DOI:** 10.1186/1471-2458-10-289

**Published:** 2010-05-28

**Authors:** Parvin Rahnama, Alireza Hidarnia, Farkhondeh Amin Shokravi, Anoushiravan Kazemnejad, Deborah Oakley, Ali Montazeri

**Affiliations:** 1 Department of Health Education, Tarbiat Modares University, Tehran, Iran; 2 Department of Biostatistics, Tarbiat Modares University, Tehran, Iran; 3 School of Nursing, University of Michigan, Ann Arbor, Michigan, USA; 4 Department of Mental Health, Iranian Institute for Health Sciences Research, ACECR, Tehran, Iran

## Abstract

**Background:**

Withdrawal as a method of birth control is still used in Iran. The aim of this study was to explore married women's perspectives and attitudes on withdrawal use instead of oral contraceptive (OC) in Tehran, Iran.

**Methods:**

This was a qualitative study. Participants were 50 married women, not currently pregnant, not desiring pregnancy and who had been using withdrawal for contraception. Face-to face interviews were conducted to collect data. Content analysis was performed to analyze the data.

**Results:**

Four major themes were extracted from the interviews: advantages, disadvantages, barriers for OC use, and husband-related factors. Advantages of withdrawal use were identified as: easy to use, convenient, ease of access, natural. Even those participants who had experienced unwanted pregnancy while using withdrawal, relied on withdrawal as their contraceptive method. Disadvantages of OC included concerns about side effects. Barriers related to use of OC included the need for medical advice, vaginal examination and daily use. Husband-related factors included: the husband wanted to be the primary decision maker on the number of children and that he preferred withdrawal.

**Conclusion:**

Health providers should address misunderstandings that exist about OC and highlight the non-contraceptive health benefits of OC to balance the information provided for women. We suggest that not only women but also their spouses be advised in family planning programs.

## Background

According to Iran's Demographic Health Survey (DHS) carried out in October 2000 by the Iranian Family Planning (FP) program, 73.7% of currently married women were using contraception, but only 55.9% used modern methods. Almost one in five married women, 18.4%, use OC; 17.1% rely on female sterilization; 2.7% rely on vasectomy; IUDs are used by 8.5%, condoms by 5.9%, and norplant implants by 0.5%. The proportion of couples relying on the traditional method of withdrawal was 17.8%, even though the national program did not encourage this method. In addition it was found that 18% of currently unintended pregnancies belonged to women who said they were using contraception, but 10% of them reported that they had used withdrawal before becoming pregnant [1].

Withdrawal is known to be associated with higher rate of unintended pregnancy [2]. This is an important public health concern because unintended pregnancies are associated with adverse effects including delayed prenatal care, pre-maturity and low birth weight [3]. A study revealed that among withdrawal users, one out of four women reported that they terminated a pregnancy because it was unplanned [4]. Thus it is likely that high rates of withdrawal use lead to unnecessary, even illegal and perhaps dangerous abortions; or to births that are mistimed or unwanted.

In Iran, modern methods of FP are easily available; FP services and primary health care units give free information on contraceptive methods to individuals or couples; and these units provide modern contraceptives free of charge. However despite these free services, Iran has a very high rate of withdrawal use compared with the world estimates [5]. Although some investigators recommend that governments should invest in counseling and in improving the availability and access to modern contraceptives [6], some other researchers, for instance, argued that reasons for not using any contraception go beyond a simple lack of information [7].

Previous studies on birth control in Iran have investigated demographic characteristics of women who used withdrawal [8,9]. For instance, a study showed that withdrawal users were younger, well educated and from urban areas [9]. However, despite the importance of these studies, they have been unable to detect the effects of non-demographic variables on choice of withdrawal as a method of birth control. The purpose of this study was to explore the topic using a qualitative method. In particular we were interested to investigate women's perceptions and attitudes about withdrawal and OC using in-depth interviews. We hypothesized that using withdrawal might be related to women's attitudes, social norms and perceived behavioral control and perhaps it could be explained in the context of Theory of Planned Behavior (TPB). This theory suggests that people's intention to perform an specific behavior is predicted or influenced by three mentioned determinants where attitude refers to a personal factor of like or dislike, social norms refers to an individual's perception of social pressure, and perceived behavioral control refers to a person's perceived confidence in the ability to perform a behavior [10].

## Methods

### Design and data collection

This was a qualitative study carried out in Tehran, Iran in 2009. Since women did not like to talk in front of others about contraceptive methods or marital status, data were collected in a comfortable setting using individual in-depth interviews rather than focus group discussions. The interviews were conducted in a semi-structured manner and were noted down rather than taped-recording due to women's wishes. All speeches by women were written as they were responding to our questions even if they talked about other issues. In addition to the main investigator (PR) a research fellow was present during the entire interviews and she also noted down interviews. A crosscheck was carried out to insure no important data were missing. The interview guide included why they used withdrawal. Other core topics in the interviews included advantages, disadvantages, barriers and facilitators related to withdrawal and OC use. The duration of interviews ranged from 35 to 40 minutes. In deciding on the number of individual interviews, data were considered saturated when no more codes could be identified.

### Study sample

Participants were recruited from women attending family planning clinics of public health services in the eastern district of Tehran, Iran. (The area serves as the training area for the Iran University of Medical Sciences). Recruitment of participants was based on convenient and purposeful sampling. Since the main objective of this study was to explore married women's experiences, subjects who met the following inclusion criteria were selected: married women between 15 and 49 years of age, who did not desire pregnancy, and who used withdrawal for contraception.

### Analysis

The data were analyzed using qualitative content analysis. Content analysis is a subjective interpretation of the content of textual data using a process of systematic classification [11]. This process uses mainly inductive reasoning, by which codes, categories and themes emerge from raw data under careful examination and constant comparison. Manifest content consists of respondents' actual words forming codes and categories, while themes are seen as expressions of the latent content [12]. Similarly for this study a qualitative content analysis was used to derive codes, categories and themes from the data, which were identified from interviews. Researcher and her coworker coded the interviews individually and over 80% of the codes were shown to be stable between the two researchers for the first five interviews, held during the first two days. Emerging categories and themes were discussed and two investigators (the main investigator and her co-worker) agreed on final themes.

### Ethics

An oral informed consent was obtained from each participant prior to the interview. Approval for the study was obtained from the Office for Protection of Research Subjects in Tarbiat Modares University.

## Results

In all 50 women were agreed to be interviewed. The demographic characteristics of the study participants are presented in Table 1. Four major themes were extracted from analysis of the data: advantages, disadvantages, barriers for OC use, and husband related factors.

**Table 1:** Demographic characteristics of the study sample (n = 50)

Age groups (years)	No. (%)
19-25	12 (24)

26-30	15 (30)

31-35	14 (28)

36-45	9 (18)

Mean (SD)	30.7 (6)

Range	19-45

**Time since marriage (years)**	

13-20	18 (36)

21-24	16 (32)

25-28	16 (32)

Mean (SD)	8 (4)

Range	13-28

**Number of children**	

One	20 (40)

Two	28 (56)

Three	2 (4)

Mean (SD)	1.6 (0.5)

Range	1-3

**Withdrawal duration (years)**	

1-4	18 (36)

5-9	21 (42)

10-13	11 (22)

Mean (SD)	6.1 (4.4)

Range	1-13

**Education**	

Primary/secondary	36 (72)

Higher	14 (18)

	

**Experience of unwanted pregnancy**	

No	41 (82)

Yes	9 (18)

**Contraceptive methods used while experienced unwanted pregnancy (n = 9)**	

Condom	2 (22)

OC*	2 (22)

Withdrawal	5 (56)

* OC: oral contraceptive

### Advantages

1. Withdrawal: All participants thought that withdrawal was a completely natural method; and a very safe approach since they did not need to take anything orally.

This is the best because it is a natural way and I don't need to take anything. It doesn't harm. Anyway, any natural things are better! (33-year-old, secondary high school, housewife, 2 children)

A woman indicates that:

We did not go to see the doctor, is natural and does not require sophisticated services, is easy to learn. (23-year-old, secondary high school, housewife, 1 child)

Most participants were satisfied with the withdrawal as indicated in their statements:

It is safe and I enjoy withdrawal while I have sex with my husband. I do not like OC because when I use pills usually I get sick.(28-year-old, secondary education, housewife, 1 child)

2. OC: A few respondents acknowledged regularity of menstruation as advantage of OC:

The pills regulate the menstruation. Once that I had irregular menses somebody told me if you take the pill, your menstruation will be tidied-up. I used for couple of months and then I get results. (30-year-old, employment, university, 2 children)

### Disadvantages

1. Withdrawal: Although most interviewees considered withdrawal a natural method, a number of respondents indicated disadvantages for withdrawal. Some participants reported that they were anxious about unwanted pregnancy during withdrawal use. Even, some women had experienced pregnancy while using withdrawal. Despite their experiences they were still eager to use withdrawal.

Every first month I am worry that whether I will get menses or not. Until it happens I think I might get pregnant. After every intercourse I ask from my husband, are you sure you were careful? (32- year-old, secondary high school, housewife, 2 children)

2. OC: Women were unhappy to use OC. They believed that OC was an unnatural method.

The pills are chemicals that go into our body. They are very harmful. I have not heard any benefits for pills. Even, when I become ill, I wouldn't like to take tablets! I prefer to use withdrawal method instead of using the pill. Is it right? (29-old-year-secondary high school, housewife, 2 children)

In addition, misunderstanding and concerns about side-effects of OC were exaggerated by respondents. A few participants stated that OC caused hypomenorrhea. Women attributed different health problems to decreased bleeding amount during menstruation or amenorrhea. A few women considered wet cupping (hijama) similar to menstruation as in both (hijama and menstruation) dirty blood could be released from the body. They believed that the occurrence of menstrual bleeding was a sign of being healthy. The participants viewed that; if the bleeding amount during menstruation decreases or the women does not menstruate the dirty blood would accumulate in the uterus and cause health problems. Hijamah (also Al-hijama) is derived from the Arabic word hajm that means, "sucking". Among Muslims, hijamah typically refers to the traditional medicine practice of wet cupping, or bloodletting with the aid of suction cups. This practice was highly encouraged by the Prophet Mohammad (peace be upon him). Hijamah continues to be practiced by Muslims today.

*I used pill for two to three months and my bleeding was decreased. It made me worry. I was telling to myself, if I continue the pill maybe I will have not menstruation. During that time I was so bad and have tiredness and weakness feeling because I was taught that the bleeding of menstruation works as venosuction to get out dirty blood. So during every menstruation I have good feelings. (24-year-old, secondary high school, housewife, 1 child*)

Infertility was mentioned as an important threat of OC. Women thought that the ovaries would be weakened while using OC.

A couple of months after taking the pill my menstruation bleeding were decreased. Someone told me that pill makes ovaries lazy. I was scared that I never could get pregnant. Certainly I don't want to have any more children but I wouldn't like to be infertile. (27-year-old, secondary high school, housewife, 2 children)

Another participant pronounced:

Before the first pregnancy I never used the pill because it makes ovaries sluggards so a lady never can get pregnant. (27-year-old, university, employee, 2 children)

Another misconception related to OC that provide a negative attitude was fear of ineffectiveness.

A respondent said that:

I heard that the people who use the pill they scare from pregnancy even they visit the doctor or take laboratory tests but they don't have confidence. (29-year-old, high school, housewife, 3 children)

Weight gain was another reason for not using OC. A woman said that:

After my second delivery, for almost one year I took pills. I got obese and my tummy became big. My husband told me this is due to pills. Because the obesity bothered me, I did not take pills any more. (29-year-old, secondary high school, housewife, 1 child)

### Barriers for OC use

Most women indicated several reasons for not using OC including need for medical advice, vaginal examination and daily use. For example, women mentioned vaginal examination is associated with the experiences of pain and embarrassment.

I hate the vaginal exam; it is painful. I am ashamed to do it. Certainly, its pain is not too much, but it is too hard. (38-year-old, university, employee, 2 children)

The majority of participants said that OC need daily intake and one should not forget to take the pill on a daily basis. They worried that they might forget to take the pill everyday, resulting in an unwanted pregnancy.

I know that I have to take the pill on time and every night. If I do forget then I will get pregnant. I always fear! I used the pill on a regular basis but the withdrawal doesn't need anything that I forgot and is so easy. (32-year-old, secondary high school, housewife, 3 children)

### Husband related factors

Women described that their husbands wanted to be primary decision maker for the number of children and preferred withdrawal use.

My husband disagrees with the use of the oral pill until we could have two children. (26-year-old, primary school, housewife, 1 child)

Another woman said:

When I got married, my husband told me that withdrawal is better. However, later I understood that his preference was due to the fact that he wanted to have a power to control the childbearing time and the number! (39-year-old, secondary high school, housewife, 2 children)

Another important factor causing husbands' imposition was that they thought that OC would damage the women's health.

A participant said:

My husband said, I am easy with this method (withdrawal). Pill is unsafe for you. It makes you infertile and obese. Possibly you will get pregnant, but I am completely in control. (36-year-old, secondary high school, housewife, 1 child)

## Discussion

Although not in depth, this study provides useful information on withdrawal and OC use from Iran. Apparently there were many mistaken perceptions about withdrawal and OC use among Iranian women. All participants believed that the withdrawal was a natural method. The most common reason for using withdrawal was the fact that women believed this method had no side effects. This idea was shared by men as well. Similarly in a study carried out to understand men's perspectives on withdrawal use, among both users and non-users in Turkey, one of the advantages reported was being free from side effects [13].

OC are known to affect menstruation, which was considered a disadvantage for using OC. Some women believed that menstrual bleeding and hijama could release dirty blood from the body. In this study hypomenorrhea was considered harmful by women. In India and Turkey there are also beliefs related to the idea that during menstruation dirty blood leaves the body [14,15]. Another study reported that women believed amenorrhea was associated with many problems including fatigue, head-aches, black or dark faces, gray skin, weakness and weight loss [16]. Scott and colleagues identified that concerns about hormonal contraception included misconceptions such as: contraception stunts growth, causes infertility, contains harmful chemicals, and causes cancer [17]. Other investigators also reported about such concerns. For instance, Guendelman and colleagues found that 70% of Latinas (compared to 41% of non-Latinas) were ambivalent about the safety of OC [18].

Most women in this study mentioned only disadvantages for using the OC. There was no weighting of risks versus benefits since the vast majority of withdrawal users saw no benefits for OC. Only two participants were able to recognize the non-contraceptive health benefits for OC. A study in suburban Turkey also revealed that women were unaware of the non-contraceptive benefits of modern methods except for condom [15]. Advantages and disadvantages regarding withdrawal and OC use, seems could be explained within the context of Theory of Planned Behavior (TPB) [19]. It is argued that TPB is a useful model that could explain and predict the intention and OC behavior among women [20]. In fact we believe the findings from this study have a potential to be explained in the framework of this theory where women's attitude was found to be positive about withdrawal, and influenced by their husbands' preferences (social norms) and indicated several perceived barriers for using OC (perceived behavioral control).

This study recognized that respondents thought that they did not need to receive counseling from health care providers for withdrawal use and most women obtained their information concerning oral pills through social networks. These findings were not unexpected, since others have reported similar findings. A study from Turkey showed that informal sources were prevalent, particularly among non-users of contraception methods [21]. A study in rural Kenya also indicated that social networks influenced contraceptive use and these social networks provided information mainly through social learning [22]. However, health providers should be aware that for many women social networks are the main source of information. Perhaps peer education might be a useful strategy for delivering information in such communities. In addition it is important to provide a supportive environment that allows women to discuss their beliefs with health personnel when attending health care centers for counseling. It seems that improving provider-client communication, providing more reliable sources, giving written take-home information, and even home visits should be considered in order to contribute to women's empowerments and increased informed choices about pregnancy. A population-based study from France found that contraceptive discontinuation rates among French women were low and concluded that this might be attributed to the role of healthcare providers in helping women to make right decision [23].

Vaginal examination mentioned as a barrier related factor to OC use and was associated with the experiences of pain and embarrassment. These concerns are not unique. Peremans et al. found that factors inhibiting visits for obtaining contraceptives were the cost, waiting time and fear of gynecological examination [24]. However, there is no evidence to support routine vaginal examination either for women starting hormonal contraception or for monitoring long term use of OC [25]. A study from the USA (where pelvic examination has been regarded as m**a**ndatory) demonstrated a clear benefit when pelvic examination was omitted from routine assessment of women attending for FP advice [26].

Finally, we found that women felt their husbands wanted to make decisions about the number of children, and preferred withdrawal as their method of choice. Husbands who wanted more children than did their wives had a higher level of withdrawal use, reflecting an enhanced desire to retain control of reproductive decision-making. This is consistent with the argument that men sometimes use withdrawal as a way to reinforce their decision-making and sexual control [27]. It has been suggested that gender-based power relations can have a direct effect on the ability of partners to acquire information relevant to their reproductive health, on their ability to make decisions related to their health, and on their ability to take action to protect or improve their health [28]. However, Kulczycki stated that measures of 'male authority' in using withdrawal have only partial predictive power in the analysis after controlling for other variables [29]. Similarly a study on the influence of gender context in five Asian countries about husbands' versus wives' fertility goals and use of contraception found that husbands' pronatalism contributes a relatively small degree to wives' contraception unmet needs [30].

## Limitations

This study has the inherent limitations due to the fact that the interviews were not recoded. The main issue was that the analysis remained at a very descriptive level. Future studies from Iran should take into account sociological and demographical characteristics into account to study women's practices and representations. In addition the husbands' implications should be much more developed and discussed in a gender perspective. For instance, in what kind of relationships was the husbands' views more likely to be taken into account, and what did the women think about it? Furthermore the different levels of analysis is recommended: were the women who feared side effects were the same as those who let their husband responsible for the choice of the method, and why?

## Conclusion

Contraceptive counseling should stress accurate information and address any misconceptions women may have about the safety of oral contraceptives. We suggest that not only women but also their spouses be advised in family planning programs because they may influence the women's contraceptive behavior, or they may support women using a safe method of contraceptive.

## Competing interests

The authors declare that they have no competing interests.

## Authors' contributions

PR was the main investigator, designed the study, collected the data, performed analysis and wrote the first draft. AH supervised the study. FA, and AK, were the study advisor. DO contributed to drafting the paper. AM critically evaluated the paper, responded to reviewers' comments, and provided the final draft. All authors read and approved the final version of the manuscript.

## Pre-publication history

The pre-publication history for this paper can be accessed here:

http://www.biomedcentral.com/1471-2458/10/289/prepub
